# Non-rigid registration of breast surfaces using the laplace and diffusion equations

**DOI:** 10.1186/1475-925X-9-8

**Published:** 2010-02-12

**Authors:** Rowena E Ong, Jao J Ou, Michael I Miga

**Affiliations:** 1 Department of Biomedical Engineering, Vanderbilt University, Nashville, TN, USA

## Abstract

A semi-automated, non-rigid breast surface registration method is presented that involves solving the Laplace or diffusion equations over undeformed and deformed breast surfaces. The resulting potential energy fields and isocontours are used to establish surface correspondence. This novel surface-based method, which does not require intensity images, anatomical landmarks, or fiducials, is compared to a gold standard of thin-plate spline (TPS) interpolation. Realistic finite element simulations of breast compression and further testing against a tissue-mimicking phantom demonstrate that this method is capable of registering surfaces experiencing 6 - 36 mm compression to within a mean error of 0.5 - 5.7 mm.

## Background

As breast cancer is estimated to kill over 40,600 people and be diagnosed in more than 194,000 in 2009 [1], its detection and treatment is an important area of scientific research. Many novel techniques to aid in tumor detection are being developed that exploit the difference in physical properties between healthy and cancerous tissue. Some of these techniques measure the optical, electrical, or elastic properties of tissue, such as near-infrared tomography [2], electrical impedance tomography [3], ultrasound elastography [4], magnetic resonance elastography [5,6], and modality-independent elastography (MIE) [7-9]. Regardless of the means of data acquisition, it is important to recognize and account for the soft-tissue deformation mechanics of the breast during any analysis.

Previous work in non-rigid registration methods can be broadly categorized as being feature-based or intensity-based. Feature-based methods use only the geometric information extracted from an image, such as a polygonal mesh constructed from a segmented image. Examples of feature-based methods include symmetric closest point [10], robust point matching [11], methods involving implicit functions [12], and finite element modeling [13]. One type of feature-based registration involves the use of splines to interpolate the displacements between tracked control points. Polynomial splines, B-splines, and thin-plate splines (TPS) are among the most commonly used. However, the difficulty with using any type of spline is determining accurate displacements at the control points; the displacements must either be tracked with fiducial markers or estimated using another method such as in [14].

In contrast, intensity-based methods utilize the intensities in the image volume, sometimes in addition to the geometric information, to register two images. Rueckert [15,16] proposed a method to maximize image similarity and preserve smoothness, while a similar volume-preserving optimization method was developed by Rohfing [17]. Tanner [18] employed a free-form b-spline deformation to maximize image similarity in a volume-preserving cost function, similar to Rueckert. The registration algorithm was validated by taking clinical dynamic contrast enhanced MR images, deforming them using a biomechanical FEM model and then calculating a TRE. Optical flow [19] and fluid flow [20] techniques have also been used for breast image registration. Unfortunately, like all optimization schemes, intensity-based methods are subject to the need for good initialization and vulnerable to local minima. In addition, intensity information may not be readily available for some applications such as near-infrared breast tomography, electrical impedance tomography, microwave tomography, or mechanical imaging. Even if intensity images are available, they may be subject to geometric distortions or contrast changes.

For the particular application of registering breast surfaces having undergone a quasi-static mechnical compression, our prior work has indicated that fiducial-based spline interpolations, while powerful, do not translate well with experimentation on tissue and substances not amenable to the fixation of physical markers. Likewise, intensity-based methods may not be desirable due to computational expense or the unavailability of suitable intensity images. Therefore, in an attempt to balance the best attributes of these classes of methods, we present a semi-automated method that does not rely on either control points or explicit knowledge of the internal image intensity pattern. This is accomplished by using the Laplace or diffusion equations to calculate equivalent surface energy distributions in order to estimate a generalized displacement field. The accuracy of the method was evaluated by comparison to our internal gold standard of a thin-plate spline interpolation method [21] in both biomechanical simulations and experimental deformations on breast phantoms.

## Methods

### PDE-based registration

The basic premise of this work was to evaluate whether the equivalent potential energy distributions modeled by a partial differential equation (PDE) over an undeformed ("source") surface and a deformed ("target") surface could be used to determine correspondence between the two surfaces. For our specific method, finite element models of two classic PDEs (Laplace's equation and the diffusion equation) were solved independently over the source and target surfaces using the Galerkin method of weighted residuals with Lagrange polynomial interpolation [22]. Equivalent potential energy isocontours were calculated and matched on a closest-point basis, and from this correspondence the final displacements at all mesh nodes were interpolated.

Laplace's equation is most commonly used to describe potential flow problems in thermal, fluid, and electrostatic systems and is given by(1)

where Φ represents the potential and σ describes the spatially varying conductivity. The diffusion equation, which utilizes a time-varying component, is given by(2)

where Φ represents the potential and *α *is the diffusion coefficient.

**Figure 1:**
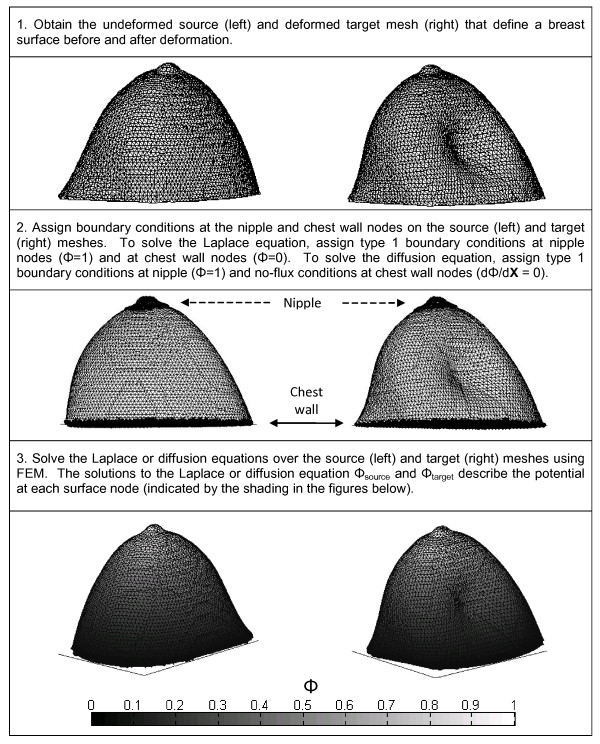
**Summary of the PDE-based registration methods (steps 1-3)**.

To solve Laplace's equation (1), Dirichlet (Type I) boundary conditions were set to allow "flow" from a high- to low-potential area (Figure 1, Step 2). Specifically, nodes in the nipple and chest wall area were given boundary potential values of 1 and 0, respectively, and the conductivity *σ *was set to unity. The diffusion equation (2) was solved by temporally stepping the FEM solution using a fully explicit forward Euler scheme. For each data set utilized in this study, optimal ranges were empirically determined for time step ([1e-7, 8e-7]) and final time ([0.005, 0.01]). A pure Neumann (Type II, no flux) boundary condition was prescribed at the chest wall, and the potential field was allowed to propagate from a source located at the nipple. The diffusion coefficient *α *was set to unity. The diffusing front was stopped once the potential field reached the chest wall.

**Figure 2:**
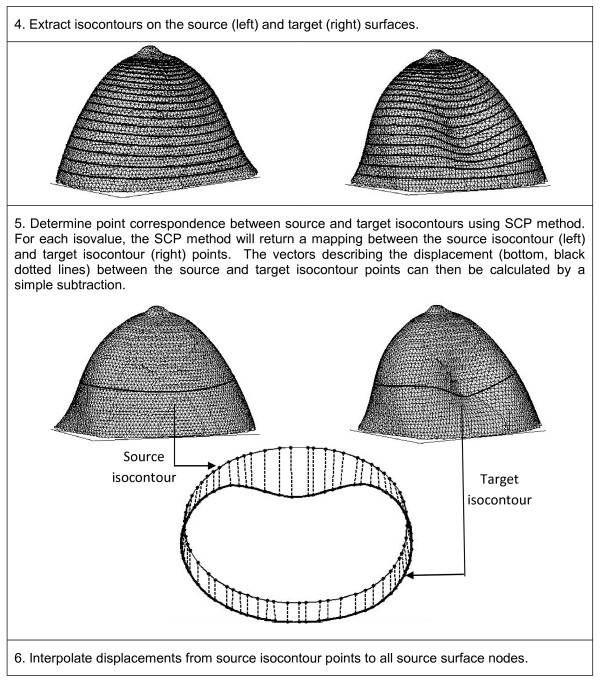
**Summary of the PDE-based registration methods (steps 4-6)**.

**Figure 3:**
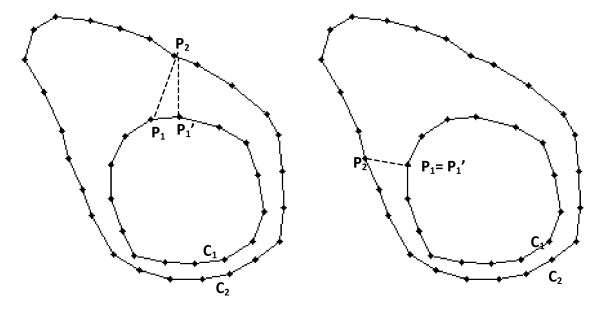
**Symmetric Closest Point algorithm description**. The symmetric closest point algorithm finds correspondence between the points in two contours by first finding the set of symmetric closest points, then using that set to find remaining point correspondence. In the left illustration above, P1 and P2 are not symmetric closest points; in the right illustration, P1 and P2 are symmetric closest points. To find the symmetric closest points for each point P1, the nearest neighbor P2 on contour c2 is found, and for each P2, the nearest neighbor P1' on C1 is found. If P1 = P1', then P1 and P2 are considered symmetric closest points.

The solutions Φ_source _and Φ_target _obtained from the PDEs were used to establish correspondence between the source and target nodes. This involved two distinct processes: finding point correspondence between isocontours of Φ_source _and Φ_target _and interpolating the displacements at these isocontour points to all nodes in the mesh. In the first step, isocontours were extracted from Φ_source _and Φ_target _for a set of selected isovalues (Figure 2, Step 4). The correspondence between the source and target isocontour points was determined by aligning the contours by their centroids and using the symmetric closest point (SCP) algorithm (Figure 2, Step 5. See Figure 3 for detailed description of SCP). In the second step, the displacement vectors at the source isocontours points were interpolated to all source nodes.

The method can be summarized in the following steps (Figures 1, 2):

1. Obtain the undeformed source mesh and deformed target mesh that define a breast surface before and after deformation.

2. Assign boundary conditions at nipple and/or chest wall nodes

3. Solve the PDE (diffusion or Laplace) over the source and target meshes using FEM.

4. Extract isocontours on the source and target surfaces.

5. Determine point correspondence between source and target isocontours using SCP (Figure 3).

6. Interpolate displacements at source isocontours to all source nodes.

### TPS registration

As noted earlier, there are numerous methods of spline-based interpolation. TPS interpolation was chosen in part because it is a standard, well-characterized method in the literature [20] that has been successfully used in many non-rigid registration applications. Because it does not require a regular grid, the effects of changing a control point are relatively localized. The overall registration is achieved by the warping of a hypothetical thin sheet of metal using a series of radial basis functions based on a number of fixed control points. The global deformation field was then interpolated back to the surface node coordinates of the finite element mesh. The displacement vector at point (*x*, *y*, *z*) is therefore described by the following linear system:(3)

where

with constraints

where **X**_**i**_, **Y**_**i**_, **Z**_**i **_are the coordinates of the control points, **N **is the number of control points, and **a, b, c**, and **F **are scalar weighting factors.

### Simulation experiments

To assess the accuracy of the PDE-based and TPS methods described above, breast surfaces deformed using a biomechanical model were registered and the simulated displacements were used to calculate the registration error. A CT image volume of a pendant human breast (256 × 256 × 130, voxel size 0.6 mm^3^) and an MR image volume of a pendant breast from a different patient (256 × 256 × 98, voxel size 1.0 mm^3^, 3D T1-weighted fat-nulling inversion pulse sequence) were obtained. Both image volumes were segmented using ANALYZE 6.0 (Mayo Clinic, Rochester, MN), and triangular source meshes consisting of 6,313 and 3,942 nodes, respectively, were constructed.

Target surfaces were created by deforming the source surfaces using a finite element model of the breast as a linear elastic, Hookean solid under localized compression. The nodes along the chest wall were made to be fixed (Type I) and a Gaussian-shaped stress distribution (Type II) applied to the lateral surface of the breast. To provide a challenge to our registration method, the shape and magnitude of the applied stress was varied. First, the CT source surface was deformed using a circular contact area with a maximum displacement of approximately 33 mm. In the second simulation, a rectangular contact area was used with a maximum displacement of 13 mm. Finally, in the third simulation, the MR source surface was deformed assuming a rectangular contact area and a maximum displacement of 6 mm.

In addition to running the Laplace and diffusion registrations, TPS registration was performed for further comparison. Because the accuracy of the TPS method varies based on the number and distribution of control points, two different sets of registrations were performed. In the first analysis, a uniform distribution of control points over the breast surface was selected using a k-means algorithm. The number of control points was varied, and for a particular number of control points desired, 20 different configurations were selected to account for the initial random seeding of the *k*-means clustering algorithm. In the second analysis, a high number of control points in the deformed region (the part of the surface in contact with the simulated inflation bladder) and a lower number over the rest of the surface was used. Similarly, the error was calculated for a varying number of control points and configurations.

To assess the accuracy of the registration methods, the target registration error (TRE) was calculated as the Euclidean distance between the coordinates determined by non-rigid registration and the true target points. Because of the controlled model-based deformation, every node on the surface had a known correspondence from source to target surfaces, allowing for individual TRE determinations along with evaluation of mean and max error for the surface as a whole.

### Phantom experiments

**Figure 4:**
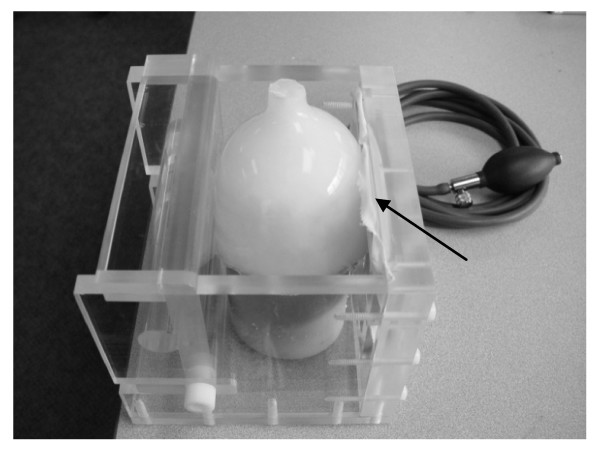
**Breast compression chamber**. Experimental system for applying compression to breast phantom. A polyvinyl alcohol cryogel is placed within a Plexiglas chamber with its surfaces held in place against the walls. Compression is delivered through an air bladder (arrow) inflated manually through a bulb adapted from a standard sphygmomanometer.

To test the registration methods on real-world data, a semi-anthropomorphic breast phantom was fabricated from an 8% w/v solution of polyvinyl alcohol (Flinn Scientific, Batavia, IL) that was frozen at -37°C in a plastic mold for 16 hours and thawed to ambient room temperature. Thirty-four 1-mm stainless steel ball bearings were implanted directly under the surface of the resulting tissue-mimicking cryogel to act as fiducials. The phantom was then placed inside a custom-built acrylic chamber designed to deliver compression by means of an air bladder positioned against the surface of the phantom (Figure 4).

CT images (512 × 512 × 174, 0.54 × 0.54 × 1 mm voxel size) were acquired with the phantom subjected to three different states of mechanical deformation (undeformed, 50% of maximum bladder pressure, and full inflation of approximately 200 mm Hg). Triangular surface meshes were obtained by segmentation of the image volumes using the surface extraction tools in ANALYZE, and the coordinates of the fiducial centroids localized. These meshes contained 8,127, 6,777, and 8,260 nodes, respectively. The Laplace, diffusion, and TPS methods were then used to register the phantom surface meshes as described above. Accuracy was assessed by calculating the TRE at the embedded fiducials. For the TPS method, 33 of the fiducials were used as control points in the interpolation and the remaining fiducial was reserved for calculating the TRE. To assess the error over the entire surface, the TPS registration was repeated in a "leave one out" scheme, each time using a different fiducial to calculate the error, with the final TRE representing the average over all trials.

## Results

### Simulation experiments

**Figure 5:**
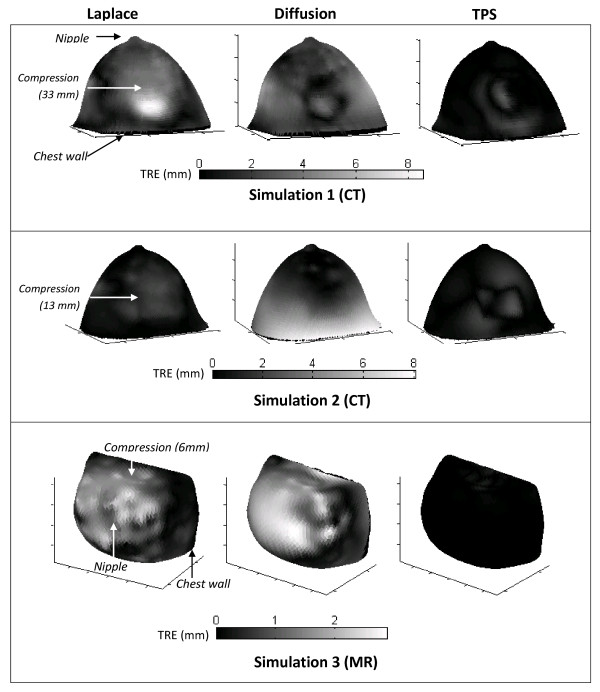
**Simulation registration error**. Error when breast surfaces deformed by three simulations were registered using the Laplace (left column), diffusion (middle column), and TPS (right column) registrations. The TPS registration method had lower error than the Laplace or diffusion methods in all three simulations.

As described in Section 2.3, the Laplace and diffusion methods were used to register the breast surfaces deformed by the three simulated compressions. For each simulation, the accuracy of the Laplace and diffusion methods was assessed by calculating the TRE at each node and comparing it with the TRE obtained using the TPS method (Figure 5). The results (Table 1) indicate that the Laplace and diffusion methods could register breast surfaces with up to 33 mm of compression with errors of 0.5 - 1.8 mm, while the TPS method generated errors of up to 0.44 mm.

**Table 1:** Simulation registration error

	Simulation 1 (CT) (33 mm displacement)		Simulation 2 (CT) (13 mm displacement)		Simulation 3 (MR) (6 mm displacement)	
	
	Max TRE (mm)	Mean TRE (mm)	Max TRE (mm)	Mean TRE (mm)	Max TRE (mm)	Mean TRE (mm)
Laplace	8.5	1.6	2.6	0.53	2.5	0.48

Diffusion	6.7	1.8	8.0	1.5	2.9	0.61

TPS*	3.1	0.44	2.6	0.26	0.6	0.033

Max and mean TRE when the Laplace, diffusion, and TPS methods were used to register breast surfaces deformed by three simulations.

*TPS registration using 40 uniformly distributed fiducials for the CT simulations and 60 for the MR simulation.

**Figure 6:**
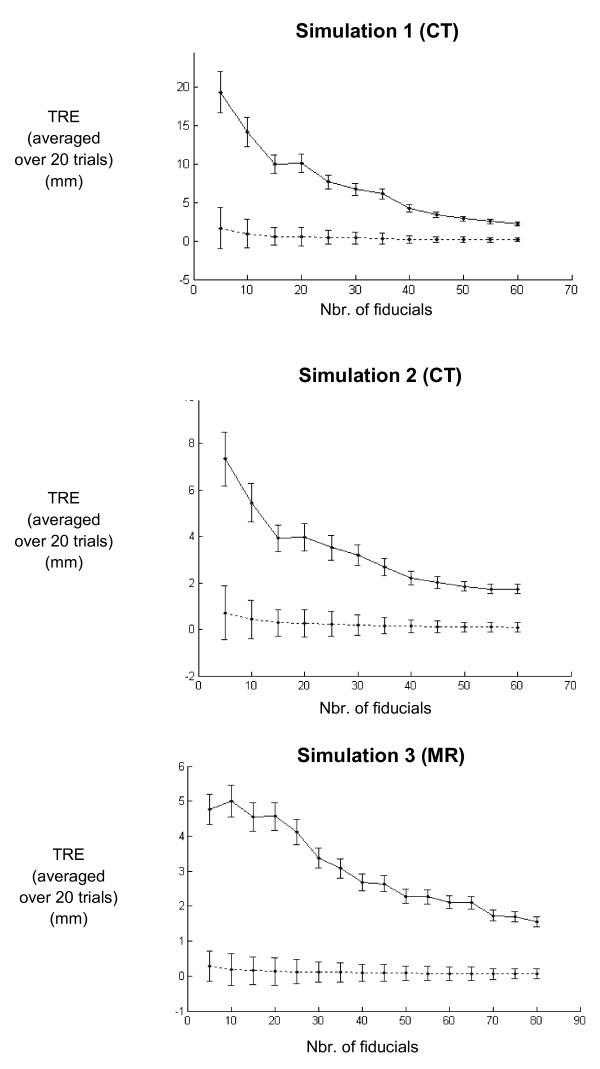
**TPS registration error for uniformly distributed fiducials**. TPS registration error (averaged over 20 trials) for breast surfaces deformed by three simulations. Max and mean TRE (solid and dotted lines, respectively) were calculated for different numbers of uniformly distributed fiducials. TPS registration error decreased as the number of fiducials increased when a uniform distribution of fiducials was used.

**Figure 7:**
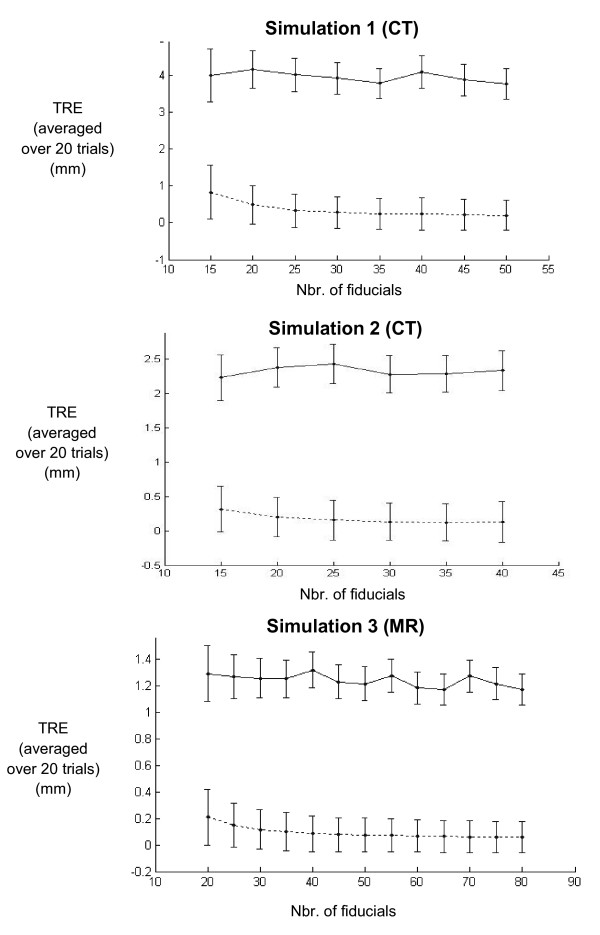
**TPS registration error for non-uniformly distributed fiducials**. TPS registration error (averaged over 20 trials) for breast surfaces deformed by three simulations. Max and mean TRE (solid and dotted lines, respectively) were calculated for different numbers of non-uniformly distributed fiducials, where a high number of fiducials was placed in the region contacting the simulated compression bladder and a varying number elsewhere. When a high number of fiducials was placed in the contact region, increasing the number of total fiducials did not significantly decrease the TPS registration error.

To evaluate how the number and placement of fiducials affects TRE, the TPS registration was performed for differing numbers of fiducials, placed in uniform (Figure 6) and non-uniform (Figure 7) fiducial distributions. The results of the TPS registrations indicate that when a uniform fiducial distribution is used, the error decreases as the number of fiducials is increased. However, increasing the fiducial number over about 40 does not seem to result in a significant error reduction. For a non-uniform distribution, the error does not seem to decrease as the number of fiducials outside the contact region is increased. In other words, the same amount of error could be obtained using a smaller number of fiducials, as long as more control points are placed in the contact region of the simulated compression bladder.

### Phantom experiment

The Laplace and diffusion methods were used to determine point correspondence between the uncompressed and compressed surfaces of a breast phantom. The results were validated by calculating the TRE at 34 fiducials located directly below the surface of the phantom. For comparison, TPS registration was used to interpolate the displacements at the fiducials to all surface nodes and the TRE was calculated as described in Section 2.4. The results for a 50 and 100% compression (with a maximum displacements of about 20 mm and 36 mm, respectively) are shown in Table 2.

**Table 2:** Phantom registration error

	Phantom: 50% compression (20 mm displacement)		Phantom: 100% compression (36 mm displacement)	
	
	Max TRE (mm)	Mean TRE (mm)	Max TRE (mm)	Mean TRE (mm)
Laplace	8.6	3.4	15.3	6.3

Diffusion	6.8	2.7	13.6	5.7

TPS *	3.4	1.1	5.1	1.7

Error for different registration methods tested on the breast phantom at 50% and 100% compression.

* TPS registration was performed using 33 fiducials and 1 fiducial to calculate TRE. The TRE was averaged over 34 trials, where each trial used a different fiducial to calculated TRE.

## Discussion

While neither the Laplace nor diffusion PDE-based methods were able to surpass the performance of a well-executed TPS-based interpolation, the results are encouraging overall as a first attempt of a method that does not require fiducials.

One area of further development with regards to the Laplace equation method is in determining specific regions to which boundary conditions are assigned. For the geometry of the breast, the nipple and chest wall areas were relatively evident and easily set; however, proper selection of these regions is important because the implicit correspondence between these regions determines the potential energy distribution and therefore correspondence for the rest of the surface.

While we do not perform a direct sensitivity analysis for boundary condition selection, previous work indicates that as long as the segmentation and resulting boundary condition error is below a certain threshold, reasonable results may be obtained. In [9], the PDE registration methods were used in a breast elastography application, and a boundary condition sensitivity analysis was performed. Reasonable results were obtained as long as the average error over all boundary condition nodes was below 0.5 voxels. Although a direct sensitivity analysis may be desirable, we feel this threshold may indicate the acceptable error for our PDE registration methods.

Some factors to consider in the diffusion method are the parameters controlling the behavior of the diffusion front over the breast surface. Careful selection of these parameters therefore enabled the diffusion-based method to outperform the Laplace method in certain cases. We believe this advantage may be related to the fact that the diffusion method requires fewer initial selections of boundary conditions to generate potential flow as compared to the Laplace method.

Although the gold standard TPS method outperformed the Laplace and diffusion methods, there are several factors to consider. The gold standard TPS interpolation method requires multiple points of constraint and is highly dependent on their number and placement. When a uniform distribution is used, the error decreases as the number of fiducials is increased but with diminishing returns. It should also be noted that to attain comparable TRE values, the non-uniform fiducial distribution requires fewer control points. The analysis of this behavior is illuminating in providing a set of benchmarks for future development of the PDE-based methods.

The literature is replete with registration methods developed for 2D and 3D mammographic applications, with a relative preponderance towards intensity-based methods. However, conventional intensity images may not be available for some applications, and even if available, they may difficult to utilize due to geometric distortion or contrast changes. The novel surface-based method we have presented may provide a suitable alternative for situations where intensity analysis is not amenable due to unavailability, computational complexity, unsuitable contrast, or imaging artifacts.

In further consideration of the accuracy of our method, we reviewed the excellent recent work presented in [18]. In this work, twelve different types of deformations were modeled ranging from 5 - 10 mm. For the optimal registration parameters, the average TRE was reported to be 0.45 mm with a maximum TRE across a series of data sets of approximately 4 - 6 mm. While our results are not directly comparable, we note that the simulations with the closest representative applied deformations of 6 and 13 mm in magnitude had a mean TRE of 0.48 to 0.53 and max TRE < 3 mm. We feel that this is reassuring that our method can perform at this level of accuracy without the prescribed use of either fiducials or image intensity.

## Conclusions

A novel surface-based non-rigid registration method has been developed for this work and compared to a relative gold standard of thin-plate spline interpolation. The results indicate that the Laplace and diffusion methods can accurately register breast surfaces that have experienced a wide range of physical deformation to within mean errors ranging from 0.5 - 5.7 mm. Although these PDE-based methods did not perform as accurately as the control, they may be viable registration techniques when fiducials are not available and image intensity comparison is not required.

## Competing interests

The authors declare that they have no competing interests.

## Authors' contributions

REO performed the simulation and phantom experiments, analyzed the data, and drafted the manuscript. JJO contributed significantly to the experimental design and acquisition of data, as well as providing valuable revisions to the manuscript. MIM was responsible for the initial conception of this project, much of the experimental design, writing the FEM modeling programs, and providing revisions to the manuscript. All authors have approved this final draft of the manuscript.

## References

[B1] Cancer facts and figures American Cancer Society 2009 http://www.cancer.org/downloads/STT/500809web.pdf

[B2] Brooksby BA Dehghani H Pogue BW Paulsen KD Near-infrared (NIR) tomography breast image reconstruction with a priori structural information from MRI: algorithm development for reconstructing heterogeneities IEEE Journal of Selected Topics in Quantum Electronics 2003 9 199 209 10.1109/JSTQE.2003.813304

[B3] Cherepenin V Karpov A Korjenevsky A Kornienko V Mazaletskaya A Mazourov D Meister D A 3D electrical impedance tomography (EIT) system for breast cancer detection Physiological Measurement 2001 22 9 18 10.1088/0967-3334/22/1/302 11236894

[B4] Ophir J Cespedes I Ponnekanti H Yazdi Y Li X Elastography - a quantitative method for imaging the elasticity of biological tissues Ultrasonic Imaging 1991 13 111 134 10.1016/0161-7346(91)90079-W 1858217

[B5] McKnight AL Kugel JL Rossman PJ Manduca A Hartmann LC Ehman RL MR elastography of breast cancer: preliminary results AJR Am J Roentgenol 2002 178 6 1411 1417 12034608 10.2214/ajr.178.6.1781411

[B6] Sinkus R Tanter M Xydeas T Catheline S Bercoff J Fink M Viscoelastic shear properties of *in vivo *breast lesions measured by MR elastography Magn Reson Imaging 2005 23 2 159 165 10.1016/j.mri.2004.11.060 15833607

[B7] Miga MI A new approach to elastography using mutual information and finite elements Physics in Medicine and Biology 2003 48 467 80 10.1088/0031-9155/48/4/304 12630742

[B8] Washington CW Miga M Modality independent elastography (MIE): a new approach to elasticity imaging IEEE Transactions on Medical Imaging 2004 23 1117 28 10.1109/TMI.2004.830532 15377121

[B9] Ou JJ Ong RE Yankeelov TE Miga MI Evaluation of 3D modality-independent elastography for breast imaging: a simulation study Physics in Medicine and Biology 2008 53 1 147 163 10.1088/0031-9155/53/1/010 18182693

[B10] Papademetris X Sinusas AJ Estimation of 3-D left ventricular deformation from medical images using biomechanical models IEEE Transactions on Medical Imaging 2002 21 7 786 800 10.1109/TMI.2002.801163 12374316

[B11] Chui HL Rangarajan A A new point matching algorithm for non-rigid registration Computer Vision and Image Understanding 2003 89 2-3 114 141 10.1016/S1077-3142(03)00009-2

[B12] Dinh HQ Implicit Shapes: Reconstruction and Explicit Transformation PhD Dissertation, 2002 Georgia Institute of Technology, College of Computing

[B13] Roose L Mollemans W Biomechanically based elastic breast registration using mass tensor simulation Medical Image Computing and Computer-Assisted Intervention - Miccai Pt 2 2006 4191 718 725 full_text 10.1007/11866763_88 17354836

[B14] Fei BW Duerk JL Sodee DB Wilson DL Semiautomatic nonrigid registration for the prostate and pelvic MR volumes Academic Radiology 2005 12 7 815 824 10.1016/j.acra.2005.03.063 16039535

[B15] Rueckert D Hayes C Non-rigid registration of breast MR images using mutual information Medical Image Computing and Computer-Assisted Intervention - Miccai 1998 1496 1144 1152

[B16] Rueckert D Sonoda LI Nonrigid registration using free-form deformations: Application to breast MR images IEEE Transactions on Medical Imaging 1999 18 8 712 721 10.1109/42.796284 10534053

[B17] Rohlfing T Maurer CR Volume-preserving nonrigid registration of MR breast images using free-form deformation with an incompressibility constraint IEEE Transactions on Medical Imaging 2003 22 6 730 741 10.1109/TMI.2003.814791 12872948

[B18] Tanner C Schnabel JA Quantitative evaluation of free-form deformation registration for dynamic contrast-enhanced MR mammography Medical Physics 2007 34 4 1221 1233 10.1118/1.2712040 17500454

[B19] Froh MS Barber DC Piecewise-quadrilateral registration by optical flow - Applications in contrast-enhanced MR imaging of the breast Medical Image Computing and Computer-Assisted Intervention - Miccai Pt 2 2006 4191 686 693 full_text 10.1007/11866763_84 17354832

[B20] Crum WR Tanner C Anisotropic multi-scale fluid registration: evaluation in magnetic resonance breast imaging Physics in Medicine and Biology 2005 50 21 5153 5174 10.1088/0031-9155/50/21/014 16237247

[B21] Goshtasby A Registration of Images with Geometric Distortions IEEE Transactions on Geoscience and Remote Sensing 1988 26 1 60 64 10.1109/36.3000

[B22] Lapidus L Pinder GF Numerical Solution of Partial Differential Equations in Science and Engineering 1982 Wiley-Interscience

